# Molecular characterization of dengue virus reveals regional diversification of serotype 2 in Colombia

**DOI:** 10.1186/s12985-019-1170-4

**Published:** 2019-05-08

**Authors:** Katherine Laiton-Donato, Diego A. Alvarez, Dioselina Peláez-Carvajal, Marcela Mercado, Nadim J. Ajami, Irene Bosch, José A. Usme-Ciro

**Affiliations:** 1 Grupo de Virología, Dirección de Redes en Salud Pública, Instituto Nacional de Salud, Avenida Calle 26 N° 51-20 CAN, Bogotá DC, Colombia; 2 Dirección de Vigilancia y Análisis del Riesgo en Salud Pública, Instituto Nacional de Salud, Bogotá DC, 111321 Colombia; 3 Alkek Center for Metagenomics and Microbiome Research, Baylor College of Medicine, Houston, TX 77030 USA; 4 Institute for Medical Engineering and Science, Massachusetts Institute of Technology, Cambridge, MA 02142-1601 USA; 5 Current Address: Centro de Investigación en Salud para el Trópico - CIST, Facultad de Medicina, Universidad Cooperativa de Colombia, Troncal del Caribe Sector Mamatoco, Santa Marta, Colombia

**Keywords:** Dengue virus, Molecular characterization, Phylogeny, Envelope, Evolution

## Abstract

Dengue is hyperendemic in Colombia, where a cyclic behavior of serotype replacement leading to periodic epidemics has been observed for decades. This level of endemicity favors accumulation of dengue virus genetic diversity and could be linked to disease outcome. To assess the genetic diversity of dengue virus type 2 in Colombia, we sequenced the envelope gene of 24 virus isolates from acute cases of dengue or severe dengue fever during the period 2013–2016. The phylogenetic analysis revealed the circulation of the Asian-American genotype of dengue virus type 2 in Colombia during that period, the intra-genotype variability leading to divergence in two recently circulating lineages with differential geographic distribution, as well as the presence of nonsynonymous substitutions accompanying their emergence and diversification.

**Electronic supplementary material:**

The online version of this article (10.1186/s12985-019-1170-4) contains supplementary material, which is available to authorized users.

## Main text

*Dengue virus* (DENV) is the etiological agent of dengue fever, one of the most important vector-borne viral diseases in terms of morbidity and mortality, according to the World Health Organization (WHO) [1]. In tropical and subtropical regions, there are around 3.6 billion people susceptible to DENV infections. Annually, between 50 and 200 million people are infected worldwide, of which 500,000 progress to severe dengue (SD) and more than 20,000 cases are fatal [2]. After DENV re-emergence in the 1970s and 1980s [3], Colombia has been considered a hyperendemic country with the presence of the four DENV serotypes, and a cyclic behavior of endemic/epidemic phases with peaks approximately every three to five years [4]. The appearance of severe dengue in Colombia in 1989 coincided with the expansion of the Asian/American genotype of DENV-2 throughout the Americas and the displacement of the American genotype that had been circulating since the early 1970s [5]. During the last two dengue epidemics (2010 and 2013), unprecedented numbers of dengue cases reached 157,152 and 127,219, respectively, followed by interepidemic years in which the number of cases significantly dropped [6]. Intriguingly, the mortality rate of severe dengue cases in Colombia showed a gradual increase since 2007, which was only partially reduced during 2017 and 2018.

The determinants of DENV pathogenesis and disease outcome are multifactorial. The immunologic component as well as the lack of early medical attention have been considered the main factors associated with disease progression and case fatality. However, increasing in vitro, in vivo, and epidemiological evidence also suggests an important role of the viral genetic background in determining the virulence [7–9]. The epidemic behavior of the Asian/American genotype contributed to the accumulation of genetic variability conforming several intra-genotype lineages [10, 11], whose importance in explaining virulence differences has been demonstrated [12, 13]. The objective of this study was to determine the genotype and evaluate the genetic diversity and phylogenetic relationship of dengue virus type 2 isolates from patients with dengue and severe dengue in Colombia, during the period 2013–2016.

We performed a retrospective analysis of 1101 archived serum samples from patients with clinical presentations of dengue and severe dengue, collected during the period 2013–2016, according to the mandatory report format of the Program for Dengue Virus Surveillance of the National Institute of Health of Colombia. These samples had been confirmed for dengue infection and serotyped following standard methods as part of the surveillance program. The present study was approved by the Technical and Ethical Committee for Scientific Research (CTIN/CEIN 7–2014 and CTIN/CEIN 23–2014) at the National Institute of Health of Colombia. The final clinical classifications were adjusted according to the Epidemiologic Surveillance System of Colombia – Sivigila, following the WHO recommendations for dengue with warning signs, dengue without warning signs, and severe dengue [14]. Serum samples were diluted 1/100 in Eagle’s Minimum Essential Medium, 200-μl aliquots were used for virus isolation in C6/36 cells and supernatants were collected after nine days post-inoculation or earlier if cytopathic effect was observed. A total of 45 samples were successfully isolated after the first or second passage and the serotype was confirmed by RT-PCR [15], 24 of which were analyzed in the present study, covering the different geographic regions of the country (Table 1). Nineteen of the selected viral strains were isolated from dengue fever patients, while the other 5 strains were isolated from severe dengue fever patients.

**Table 1:** List of Colombian DENV-2 strains included in the study and associated clinical outcome

Strain	Year	Department	Clinical classification	Age (Years)	Gender	Final Outcome	Genbank accession number
422,041	2013	Boyaca	Severe Dengue	54	F	Alive	KU878567
422,091	2013	Meta	Severe Dengue	5	M	Dead	KU878565
422,275	2013	Meta	Dengue	60	M	Alive	KU878566
422,641	2013	Cauca	Severe Dengue	28	M	Alive	KU878564
423,887	2013	Putumayo	Dengue	14	F	Alive	KU878570
424,029	2013	Arauca	Dengue	12	F	Alive	KU878568
425,334	2013	Putumayo	Dengue	22	M	Alive	KU878571
425,817	2013	Tolima	Dengue	1	M	Alive	KU878572
425,819	2013	Tolima	Dengue	7	M	Alive	KU878573
427,493	2013	Tolima	Dengue	13	F	Alive	MK016293
427,516	2013	Caldas	Dengue	11	M	Alive	KU878569
428,702	2014	Tolima	Dengue	5^a^	M	Alive	KU878575
434,321	2014	Meta	Severe Dengue	21	M	Alive	KU878574
449,308	2015	Huila	Severe Dengue	8	M	Dead	MK016294
449,418	2015	Tolima	Dengue	37	F	Alive	MK016298
449,510	2015	Putumayo	Dengue	NA	F	Alive	MK016299
450,024	2015	Huila	Dengue	5	F	Alive	KY905139
452,018	2015	Huila	Dengue	NA	F	Alive	MK016297
457,058	2016	Arauca	Dengue	51	M	Alive	KY905140
462,966	2016	Nariño	Dengue	54	F	Alive	MK016296
484,926	2016	Casanare	Dengue	NA	F	Alive	MK016291
484,975	2016	Huila	Dengue	8	F	Alive	MK016295
484,978	2016	Huila	Dengue	31	M	Alive	MK016300
484,995	2016	Norte de Santander	Dengue	33	F	Alive	MK016290

^a^months. *NA* Not available, *M* Male, *F* Female

For RNA extraction the QIAamp Viral RNA Mini kit (Qiagen Inc., Chatsworth, CA, USA) was used by following the manufacturer instructions. Amplification of the DENV envelope gene, was performed with the serotype-specific oligonucleotides as described by Domingo et al. [16], which amplify a 1797 bp fragment. PCR products were purified through the QIAquick PCR purification kit (Qiagen®, Chatsworth, CA, USA) and processed for direct sequencing by using the BigDye® terminator cycle sequencing v3.1 (Applied Biosystems, Carlsbad, CA, USA) and the ABI 3130 Genetic Analyzer (Applied Biosystems, Carlsbad, CA, USA). The electropherograms were visualized, edited and assembled through the SeqMan module of LaserGene® v8.1 (DNASTAR Inc., Madison, WI, USA.).

The sequences obtained in the present study and fifty seven sequences representing the different genotypes of DENV-2 previously deposited in GenBank, mainly those covering the genetic variability within the Asian/American genotype, were aligned and used for phylogenetic reconstruction through Bayesian inference using the MrBayes software [17], and a total of four MCMCs (three cold, one hot) were evaluated at 1000000 generations with sampling frequency every 100 generations for a total of 10,000 trees. The consensus tree was visualized through FigTree v1.4.3 http://tree.bio.ed.ac.uk/software/figtree/ and was edited in MEGA 7.0 software [18].

Based on the phylogenetic tree, different well-supported lineages were defined into the Asian/American genotype of DENV-2. Overall mean, intra-lineage and inter-lineage genetic distances were estimated through the MEGA 7.0 software by using the best nucleotide substitution model. The nucleotide and protein alignments showing variable sites and non-synonymous substitutions through the different domains of the envelope protein are depicted (Additional file 2: Figure S2 and Fig. 2, respectively).

**Fig. 1:**
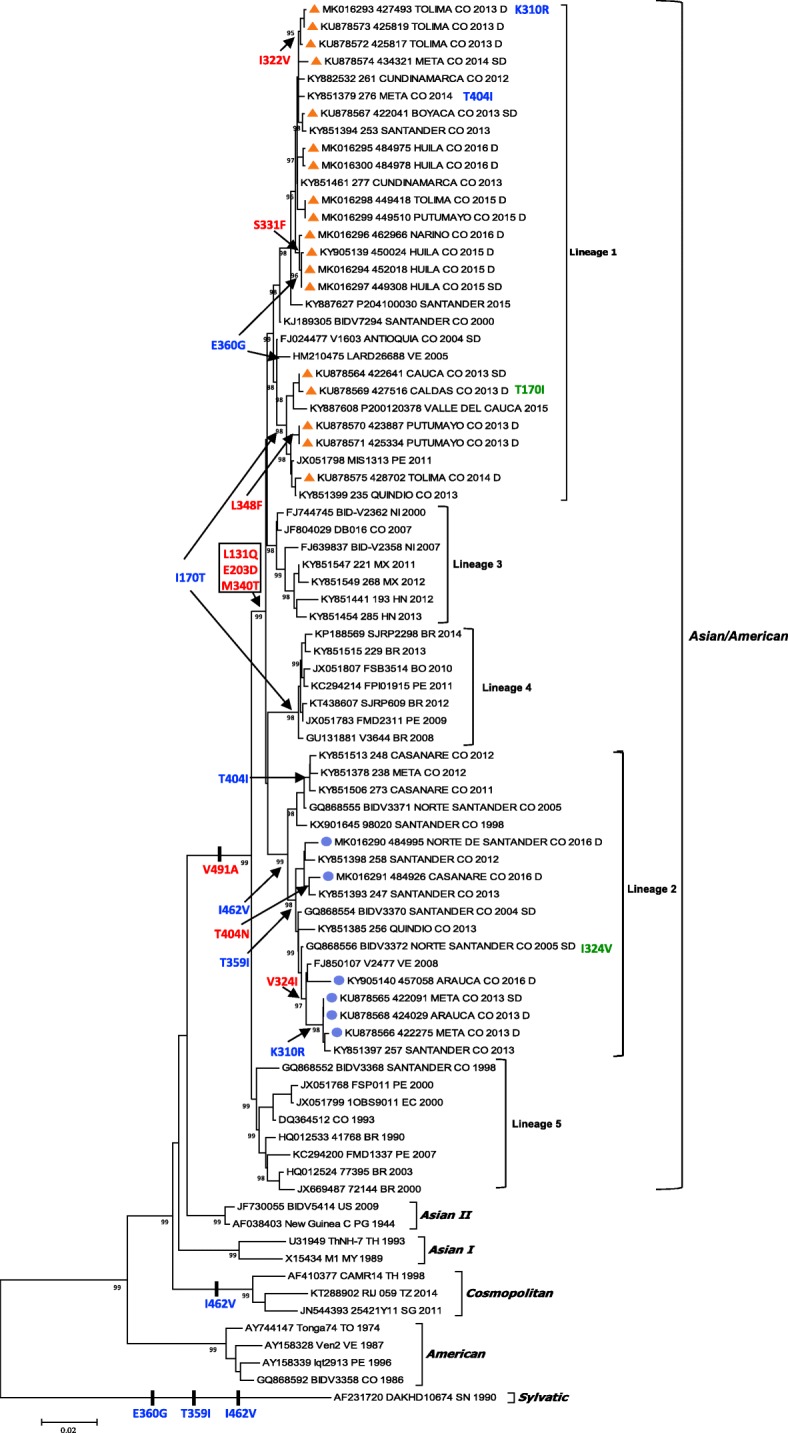
Bayesian inference of phylogenetic relationships of DENV-2 strains based on the envelope gene. The best nucleotide substitution model was GTR + I + G. Colombian strains sequenced in the present study belonging to Lineage 1 and Lineage 2 were labeled with orange triangles and blue circles, respectively. Amino acid changes in the envelope protein were mapped and depicted according to the occurrence as unique (red), convergences (blue) and reversions (green). Sequence labels included the GenBank accession number, followed by the strain name (and department in the case of Colombia), the two-letter country code (e.g. CO for Colombia, VE for Venezuela, PE for Peru, etc.) and the year of isolation

All Colombian DENV-2 strains included in the present study circulating during the period 1993–2016, belonged to the Asian/American genotype. Five well-supported intra-genotype lineages with marked spatial and temporal relationships were identified. Two of them (named Lineage 1 and Lineage 2) consisted of sequences from DENV-2 strains recently circulating in Colombia (Fig. 1). Lineages 1 and 2 were represented by sequences of strains circulating during the period 2000–2016 and 1998–2016, respectively. When estimating the global evolutionary divergence at the nucleotide level for the Asian/American genotype in the sequence alignment (using the Tamura-Nei nucleotide substitution model with proportion of invariant sites and gamma distribution with α shape = 2.9), an average of 0.031 substitutions per site was obtained between each pair of sequences of the Asian/American genotype, evidence of high intra-genotype diversity (Additional file 2: Figure S2). The estimated average evolutionary divergences within Asian/American Lineages 1 and 2 were 0.012 and 0.016 substitutions per site, respectively; while the average evolutionary divergence over sequence pairs between Lineages 1 and 2 was 0.031 substitutions per site, revealing the close relationship between strains belonging to each lineage and the marked within-country divergence of the epidemic DENV-2 strains belonging to these two lineages.

**Fig. 2:**
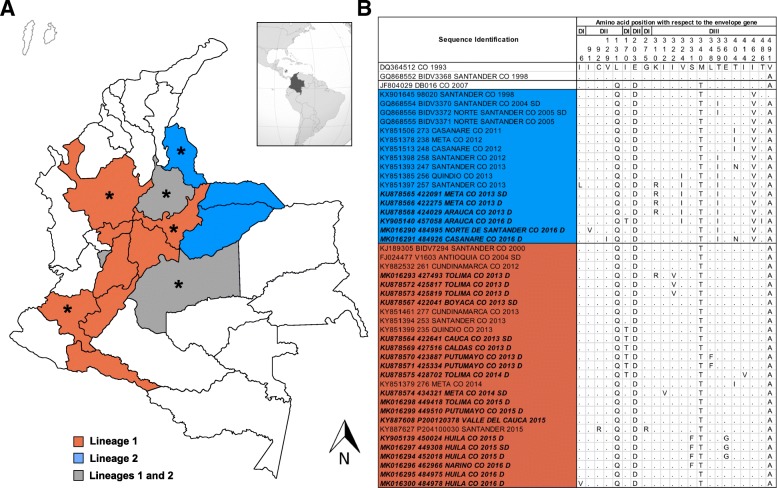
Spatio-temporal distribution of DENV-2 lineages in Colombia and accompanying amino acid changes. **a** Geographic distribution of lineages 1 and 2 of the Asian/American genotype of DENV-2 in Colombia. **b** Variation in the amino acid sequence of the envelope protein of Colombian strains of DENV-2. The amino acid sequence was inferred from the nucleotide sequences by using the standard genetic code. Representative sequences of strains that have circulated in Colombia and those obtained in the present study were aligned and variable sites along the protein sequence compared to the first Asian/American genotype from Colombia included in the analysis (Genbank accession number: DQ364512). D: Dengue; SD: Severe dengue; *: Departments where strains associated with SD were identified; DI: Domain I; DII: Domain II; DIII: Domain III

Lineage 1 was identified in the departments of Antioquia, Boyacá, Caldas, Cauca, Cundinamarca, Huila, Meta, Putumayo, Quindío, Santander, Valle del Cauca, Nariño and Tolima, that mainly encompass the Andean and Amazon regions in the Southwestern and Central portion of Colombia (Fig. 2a); while Lineage 2 was identified in the departments of Arauca, Casanare, Meta, Norte de Santander, Quindio and Santander, encompassing the Andean and mainly the Orinoquia natural regions in the East and Central portion of the country (Fig. 2a). From the analyzed dataset for the epidemic year 2013, lineages 1 and 2 co-circulated in the departments of Quindío and Santander. In the department of Meta, lineage 1 was identified in 2014 while lineage 2 was identified during 2012–2013. A recent study mainly including strains from the Santander department, allowed the identification of a single recently circulating lineage with a mean estimated time to the most recent common ancestor around 1987 and closely related to other isolates from Venezuela; however, the very low representation of sequences from the Andean and Amazon regions prevented the identification of what is denoted in the present study as Lineage 1 [19]. Lineage 1 was found to be closely related to strains from Venezuela and Peru (Fig. 1), suggesting that DENV-2 circulation in these bordering countries is marked by importation and exportation of strains, and which is supported by the geographical proximity and commercial exchange between these regions. The third lineage included strains from Central America with evidence of introduction to Colombia in 2007, but there was no evidence of dispersion and diversification inside the country. The fourth lineage included strains from Bolivia, Brazil, and Peru during the period 2008–2014, without evidence of circulation in Colombia. The fifth lineage fell in an ancestral position in the phylogenetic tree and was conformed by strains that circulated during the period 1990–2007 in Colombia and other South American countries.

When the limited information related to the clinical classification of patients from the present and previous studies was mapped to the phylogenetic tree, dengue and severe dengue cases were associated with both recently circulating lineages belonging to the Asian/American genotype (Fig. 1). All Colombian sequences obtained in the present study contained the distinctive asparagine amino acid at position 390 of the envelope protein. Twenty-two nonsynonymous substitutions were observed when Colombian sequences of the Asian/American genotype were compared to the earliest Colombian sequence included in the dataset, isolated in 1993 (Fig. 2b). Most nonsynonymous substitutions (63.6%) occurred in the domain III (residues 296–394) which has been reported to directly interact with the cellular proteins during virus entry and constitutes a major target for neutralizing antibodies [20]. An isoleucine to valine amino acid change (I312V) in the envelope protein was found to be exclusively present in one Colombian DENV-2 strain isolated from a severe dengue case in the present study. Further investigation will be needed to establish its role in viral pathogenesis.

Amino acid changes were mapped on the phylogenetic tree, enabling identification of a I462V substitution accompanying the emergence of the lineage 2 and being preserved in all descendants of the monophyletic group during the period 1998–2016. The T359I and V324I substitutions were present in lineage 2, in those strains diversifying during the period 2008–2016, as well as the K310R substitution in a subset of more recent strains belonging to a monophyletic group (2013–2016) (Fig. 1).

In contrast, the emergence of lineage 1 was not characterized by nonsynonymous changes at the envelope protein. Only a few amino acid substitutions appeared in subsets of strains. The I170T change accompanied the evolution of one strain isolated in 2011in Peru and six strains from Colombia covering the period 2013–2014. The independent occurrence of the I170T change within the lineage 1 and lineage 4 was evidence of convergent evolution. The reversion to the ancestral state (T170I and I324V) and the evidence of convergent evolution (K310R, T404I, E360G, I170T and T359I) are mainly due to amino acid changes located at domain III of the envelope protein and could be suggesting positive selection pressure acting at these sites that should be assessed in future studies.

The presence of Colombian isolates of DENV-2 through the whole branching of the highly diversified Asian/American genotype demonstrates the sustained transmission of the virus through time. The geographic and temporal segregation of the different lineages with strains from bordering countries are evidence of an intense dynamics determined by lineage extinction and a bi-directional flow of strains that could explain the drastic changes in the disease epidemiology [10, 12, 21].

The broad clinical spectrum of the disease ranging from asymptomatic to severe and fatal cases represents an opportunity for future clinical and virological studies attempting to demonstrate the existence of a viral genetic contribution to the disease outcome. The increase in the mortality rate of severe dengue cases during the last years (Additional file 1: Figure S1) suggests increased virulence of DENV strains through time. Nevertheless, unsolved difficulties in the clinical management and immunologic factors related to the hyperendemic circulation of the four serotypes or closely-related flaviviruses can also be contributing factors to the disease outcome. Under-reporting of the dengue cases to the National Surveillance System and a very low isolation rate from archived samples constitute limitations. Nevertheless, this study described the recent circulation of lineages 1 and 2 of the Asian/American genotype of DENV-2 in Colombia, the microevolution and differential geographic distribution at the national level.

Notwithstanding the growing epidemiologic and experimental data of the presence of determinants of virulence in the DENV genome [8, 22–24], further comparative analysis of full-length viral genomes and functional studies on the role of specific substitutions will be decisive for advancing the elucidation of its epidemiology and disease dynamics.

## Additional files

Additional file1: **Figure S1.** Incidence of severe dengue and mortality rate in Colombia during the period 2007–2018. The Mortality rate of severe dengue cases was estimated as the number of fatal cases per hundred severe dengue cases. * Epidemiological Week 37. (PNG 50 kb)

Additional file 2: **Figure S2.** Variation in the nucleotide sequence of the envelope gene of Colombian strains of DENV-2. Representative sequences of strains that have circulated in Colombia and those obtained in the present study were aligned and variable sites along the nucleotide sequence compared to the Asian/American genotype representative strain Jamaica N.1409. D: Dengue; SD: Severe dengue. (PDF 357 kb)

## Abbreviations

DENV: Dengue virus
RT-PCR: Reverse transcription-polymerase chain reaction
SD: Severe dengue
WHO: World Health Organization
ZIKV: Zika virus
