# Supplementary figures and images for: Molecular characterization of dengue virus reveals regional diversification of serotype 2 in Colombia
Source: Virol J. 2019 May 8;16:62. doi: 10.1186/s12985-019-1170-4 (PMC6505283; doi:10.1186/s12985-019-1170-4)

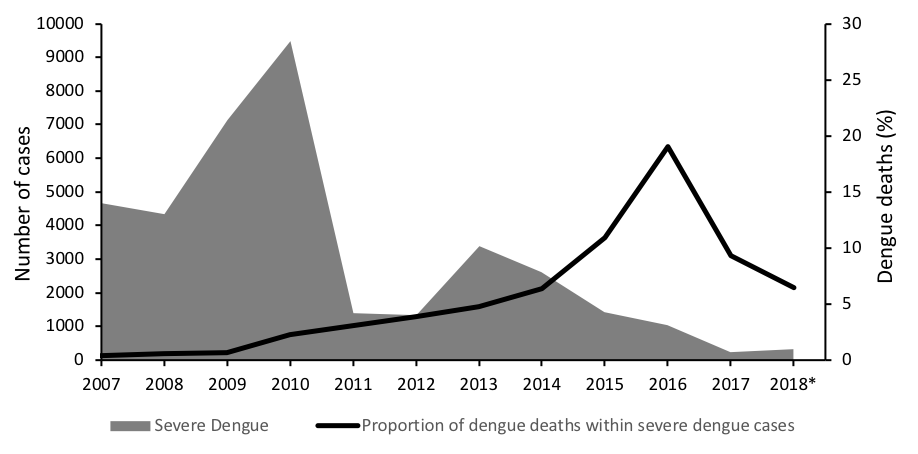

Supplement: Supplementary file 1 — Figure S1. Incidence of severe dengue and mortality rate in Colombia during the period 2007–2018. The Mortality rate of severe dengue cases was estimated as the number of fatal cases per hundred severe dengue cases. * Epidemiological Week 37. (PNG 50 kb) [file 12985_2019_1170_MOESM1_ESM.png]
