# Supplementary material for: Molecular characterization of dengue virus reveals regional diversification of serotype 2 in Colombia
Source: Virol J. 2019 May 8;16:62. doi: 10.1186/s12985-019-1170-4 (PMC6505283; doi:10.1186/s12985-019-1170-4)
Supplement: Supplementary file 2 — Figure S2. Variation in the nucleotide sequence of the envelope gene of Colombian strains of DENV-2. Representative sequences of strains that have circulated in Colombia and those obtained in the present study were aligned and variable sites along the nucleotide sequence compared to the Asian/American genotype representative strain Jamaica N.1409. D: Dengue; SD: Severe dengue. (PDF 357 kb) [file 12985_2019_1170_MOESM2_ESM.pdf]

Sequence Identification	Nucleotide position with respect to envelope gene
	111111111122222222223333333333334444444444444555555555555555666666666666677777777
	123334567890122256667001344778902233456689901224555667780001123345677990112345566679001124455
	664369546576511690258117640914216140621695293709403628143479062473213627925790141362091404767
M20558 JM/N.1409 1983	TATTAATACATGATTTTCCTAGTATGCTCTCAAATGCGTAGGTATCGTGTATCAATATCTCAATCCGGCCCGCATCCCGACACGTTGCCATTT
MK016293 427493 TOLIMA CO 2013 D	C..C....T....C.C......C.G...A.....CA...G..A.........T.G.................................T.G..C
KU878573 425819 TOLIMA CO 2013 D	C..C....T....C.C......C.G...A.....CA...G..A.........T.G.................................T.G..C
KU878572 425817 TOLIMA CO 2013 D	C..C....T....C.C......C.G...A.....CA..CG..A...........G.................................T.G..C
KU878574 434321 META CO 2014 SD	C..C....T....C.C......C.G...A.....CA...G..A...........G............A....................T.G..C
KY851379 276 META CO 2014	C..C....T....C.C......C.G...A.....CA...G..A...........G.................................T.G..C
KU878567 422041 BOYACA CO 2013 SD	C..C....T....C.C......C.G...A.....CA...G..A...........G.....................T...T......T.G..C
KY851394 253 SANTANDER CO 2013	C..C....TG...C.C......C.G...A.....CA...G..A...........G.....................T...T......T.G..C
KY882532 261 CUNDINAMARCA CO 2012	C..C....T.C....C......C.G...A.....CA...G..A...........G.................................T.G..C
MK016295 484975 HUILA CO 2016 D	C..C....T....C.C......C.G...A.....CA...G..A...........G.G............T....C............T.G..C
MK016300 484978 HUILA CO 2016 D	CG.C....T....C.C......C.G...A.....CA...G..A...........G.G............T....C............T.G..C
KY851461 277 CUNDINAMARCA CO 2013	C..C....T....C.C......C.G...A.....CA...G..A...........G..............T..................T.G..C
MK016298 449418 TOLIMA CO 2015 D	C..C..C.T....C.C......C.G...A.....CA...G..A......C....G..............T..................T.G..C
MK016299 449510 PUTUMAYO CO 2015 D	C..C..C.T....C.C......C.G...A.....CA...G..A......C....G..............T..................T.G..C
MK016296 462966 NARINO CO 2016 D	C..C....T....C.C......C.GA..A.....CA...G..A.....A.....G.......G......T..................T.G..C
KY905139 450024 HUILA CO 2015 D	C..C....T....C.C......C.G...A.....CA...G..A.....A.....G.......G......T....................G..C
MK016294 452018 HUILA CO 2015 D	C..C....T....C.C......C.G...A.....CA...G..A.....A.....G.......G......T..................T.G..C
MK016297 449308 HUILA CO 2015 SD	C..C....T....C.C......C.G...A.....CA...G..A.....A.....G.......G......T..................T.G..C
KY887627 P204100030 SANTANDER 2015	C..C....T....C.C......C.G...AC....CA...G..A.....A.....G.......G......T..................T...CC
KJ189305 BIDV7294 SANTANDER CO 2000	C..C....T....C........C.G...A.....C.......A...........G...................................G...
FJ024477 V1603 ANTIOQUIA CO 2004 SD	C..C....T....C........C.G...A.............A...........G.............T.....................G...
KU878564 422641 CAUCA CO 2013 SD	C..C....T....C........C.G...A.............A.C.........G....CT.....A.T......T..............G...
KU878569 427516 CALDAS CO 2013 D	C..C.G..T....C........C.G...A.............A.C.........G....CT.....A.T......T..............G...
KY887608 P200120378 VALLE DEL CAUCA 2015	C..CG..G.....C........CGG...A.............A...........G...........A.T......T..........A..G...
KU878570 423887 PUTUMAYO CO 2013 D	C..C....T....C........C.A...A.............A...C............CT.......T...T..T..............G...
KU878571 425334 PUTUMAYO CO 2013 D	C..C....T....C........C.A...A.............A...C............CT.......T...T..T..............G...
KU878575 428702 TOLIMA CO 2014 D	C..C....T....C........C.G...A.............A...........G....CT.......T.T.T..T..............G...
KY851399 235 QUINDIO CO 2013	C..C....T....C........C.G...A.............A...........G....CT.......T.T.T..T..............G...
KY851513 248 CASANARE CO 2012	C..CG.......GC........CGG..CA...G.........A.......G....C....T............G.....G..TT.....G.C.
KY851378 238 META CO 2012	C.CCG.......GC.......ACGG..CA...G.........A.......G....C....T............G...T.G..TT.....G.C.
KY851506 273 CASANARE CO 2011	C..CG.......GC........CGG.TCA...G.........A.......GC...C....T..........A.G.....G..TT.....G.C.
GQ868555 BIDV3371 NORTE SANTANDER CO 2005	C..CG.......GC........CGG..CA...G.........A.......G....C.................G.....G..TT.....G.C.
KX901645 98020 SANTANDER CO 1998	C..CG......CGC........CGG..CA...G.........A.......G....C.......................G..TT.....G.C.
MK016290 484995 NORTE DE SANTANDER CO 2016 D	C..CG.......GC....T...CGG..CG.............A.......GCTG.C.........T..T.........A..GT......G.C.
KY851398 258 SANTANDER CO 2012	C..CG.......GC........CGGT.CA.............AG......GC...C.........T..T.........A..GT......GCC.
MK016291 484926 CASANARE CO 2016 D	C..CG.......GC..C.....CGG..CA....G.......AA.......GC...C........TT.AT.........A..GT......G.C.
KY851393 247 SANTANDER CO 2013	C..CG.......GC........CGG..CA......A......A.......GC...C........TT..T.........A..GT......G.C.
GQ868554 BIDV3370 SANTANDER CO 2004 SD	C..CG.......GC......G.CGG..CA.............A.......GC...C.........T............A...T......G.C.
KY851385 256 QUINDIO CO 2013	C..CG.......GC........CGG..CA.T.......C...A.......GC...C..T......T............A...T......G.C.
GQ868556 BIDV3372 NORTE SANTANDER CO 2005 SD	C..CG..G....GC........CGG..CA.............A.......GC...C.........T............A...T......G.C.
KY905140 457058 ARAUCA CO 2016 D	C..CG..G....GC...T....CGG.T.A.............A..T.G..GC...C...C.G...T............A...T.C....G.C.
KU878565 422091 META CO 2013 SD	...CG.CG....GC........CGG..CA..G....T.....A........C...C.......C.T...........TA...T......G.C.
KU878568 424029 ARAUCA CO 2013 D	...CG.CG....GC........CGG..CA..G....T.....A........C...C.......C.T...........TA...T......G.C.
KU878566 422275 META CO 2013 D	...CG.CG....GC........CGG..CA..G....T.....A..T....GC...C.C.....C.T...........TA...T......G.C.
KY851397 257 SANTANDER CO 2013	.T.CG.CG....GC.....C..CGG..CA..G....T.....A.......GC...C.......C.T...........TA...T......G.C.
JF804029 DB016 CO 2007	C..C.........C........C.G...A.............A...........G..............................C...G...
GQ868552 BIDV3368 SANTANDER CO 1998	...C.........CC.............A......A........C.........................T.G....T....T.....TG...
DQ364512 CO 1993	...CG...T.C.................A......A.A......C.............T.............G.....A...........G...

Sequence Identification	Nucleotide position with respect to envelope gene
	11111111111111111111111111111111111111111111111111111111111111111
	777778888888889999999999999900000000000000000011111111112222222222222222333333333334444444444
	567892245577790011233346799901112233445566677892234557899011223344677788901113456688134445677 8
	924383865804640625903424002951490328471628699828432902476124709023235473214727628343416971232
M20558 JM/N.1409 1983	AAGCGGCTCGCCAATTAGAGAAAAGCCTCTGTGTACATCTTGCACCACCAGTCACCTCAAGGCATTCCGGTTGCATTTATACATATGACGCTG
MK016293 427493 TOLIMA CO 2013 D	.G........T...C...G....G.T.....CA.G....C.A...T.......TG..C...A.T........C...C.........C.......
KU878573 425819 TOLIMA CO 2013 D	.G........T...C........G.T.....CA.G....C.A...T.......TG..C...A.T........C...C.........C.......
KU878572 425817 TOLIMA CO 2013 D	.G........T...C........G.T.....CA.G....C.A...T.......TG..C...A.T........C.............C.......
KU878574 434321 META CO 2014 SD	.GT.......T..GC.......G...T.C...CA.G....C.A...T.......TG..C...A.T........C.............C.......
KY851379 276 META CO 2014	.G........T...C...........T.....CA.G....C.A...T....A.TG..CT..A.T.......CC.............C.......
KU878567 422041 BOYACA CO 2013 SD	.G........T...C................CA.G....C.A...T.......TG..C...A.T........C.............C.......
KY851394 253 SANTANDER CO 2013	.G........T...C...........T.....CA.G....C.A...T.......TG......A.T........C.............C.......
KY882532 261 CUNDINAMARCA CO 2012	.G........T...C...........T.....CA.G....C.A...T.......TG..C...A.T.....A..C.............C.......
MK016295 484975 HUILA CO 2016 D	.G........T...C...........T.....CA.G.....CA...T.......TG..C...A.T........C.............C.......
MK016300 484978 HUILA CO 2016 D	.G........T...C...........T.....CA.G....CCA...T.......TG..C...A.T........C.............C.......
KY851461 277 CUNDINAMARCA CO 2013	.G........T...C...........T.....CA.G....C.A...T.......TG..C...A.T........C.............C.......
MK016298 449418 TOLIMA CO 2015 D	.G........T...C.G.........T...C.CA.G....C.A...T.......TG..C...A.T........C.............C.......
MK016299 449510 PUTUMAYO CO 2015 D	.G........T...C.G.........T...C.CA.G....C.A...T.......TG..C...A.T........C.............C.......
MK016296 462966 NARINO CO 2016 D	.G........T...C...........TT....CA.G....C.A...T.......TG..C...A.T........C.............C.......
KY905139 450024 HUILA CO 2015 D	.G........T...C...........TT....CA.G....C.A.G.T.......TG..C...A.T........C.............C.......
MK016294 452018 HUILA CO 2015 D	.G........T...C...........TT....CA.G....C.A.G.T.......TG..C...A.T........C.............C.......
MK016297 449308 HUILA CO 2015 SD	.G........T...C...........TT....CA.G....C.A.G.T.......TG..C...A.T........C.............C.......
KY887627 P204100030 SANTANDER 2015	.G...A....T...............T.....CA......C.A...........TG..C...A.T........C...........GC.......
KJ189305 BIDV7294 SANTANDER CO 2000	.G........T...............T.....CA......C.A...........TG..C...A.T........C...........GC.......
FJ024477 V1603 ANTIOQUIA CO 2004 SD	.G........................T.....CA......C.A....G......TG..C...A.T.....................GC.......
KU878564 422641 CAUCA CO 2013 SD	.G.............C..........T.....CA.....TCCA....G...A.T.......A.T.C...................GC.......
KU878569 427516 CALDAS CO 2013 D	.G.....C.......C..........T.....CA.....TCCA....G...A.T.......A.T.C...................GC.......
KY887608 P200120378 VALLE DEL CAUCA 2015	.G.............C..........T.....CA..T..TCCA....G...A.T.......A.T.C..T................GC.......
KU878570 423887 PUTUMAYO CO 2013 D	.G........................T.....CA...C..C.A....G...A.T.......A.T...................G...GC.......
KU878571 425334 PUTUMAYO CO 2013 D	.G........................T.....CA...C..C.A....G...A.T.......A.T...................G...GC.......
KU878575 428702 TOLIMA CO 2014 D	GG........................T.....CA...G..C.A....GT..A.T.......A.TG....................G........
KY851399 235 QUINDIO CO 2013	GG........................T.....CA...G..C.A....G...A.T.......A.T.....................GC.......
KY851513 248 CASANARE CO 2012	.G.T...............A.....G.....CAC.T.....A..T...T..CTG..CT..A.......A.....G.......G.G....A...
KY851378 238 META CO 2012	.G.T...............A.....G.....CAC.T.....A......T..CTGT.CT..A.......................G.G....A...
KY851506 273 CASANARE CO 2011	.G.T...............A.....G.....CAC.T.....A......T..CTG..CT..A.......................G.G....A...
GQ868555 BIDV3371 NORTE SANTANDER CO 2005	.G.................A.....G.....CAC.T.....A......T..CTG..C...A.........C...........G.G....A...
KX901645 98020 SANTANDER CO 1998	.G.................A.....T.....CAC...C...A.........CTG..C..GA.........CC..........G.G....AT..
MK016290 484995 NORTE DE SANTANDER CO 2016 D	.G.......A.........A..G..T.C...CAC.....C.AT.........T...C...A.........CCA........TG.G........
KY851398 258 SANTANDER CO 2012	.G.......A.T.......A..G..T.....CAC.....C.AT.........TG..C...A.........CCA.........G.G......C.
MK016291 484926 CASANARE CO 2016 D	.G.......A.........A..G..T.....CAC.....C.AT......G..TG..CAC.A.........C.A.........G.G........
KY851393 247 SANTANDER CO 2013	.G.......A.........A..G..T..T..CAC.....C.AT.........TG..CAC.A.........CCA.........G.G.A......
GQ868554 BIDV3370 SANTANDER CO 2004 SD	.G.......A.........A.....T.....CAC.......AT.........TG..C...A.........C...........G.G........
KY851385 256 QUINDIO CO 2013	.G.......A.........A....AT.....CAC...C...AT....T....TG..C...A....C...AC.......T...G.GC.......
GQ868556 BIDV3372 NORTE SANTANDER CO 2005 SD	.G.......A.........A.....T.....CAC.......AT.........TG..C...A.........C.....C.....G.G........
KY905140 457058 ARAUCA CO 2016 D	.G....T.TA.........AG...AT.....CAC.......AT.........TG..C...A....CT...C.....C..C..GCG...T....
KU878565 422091 META CO 2013 SD	.G.......A........GA....AT....ACAC.......AT.........TG..C...A....C....C.....CC.C..G.G........
KU878568 424029 ARAUCA CO 2013 D	.G.......A........GA....AT....ACAC.......AT.........TG..C...A....C....C.....CC.C..G.G........
KU878566 422275 META CO 2013 D	.G.......A........GA....AT....ACAC.......AT.........TG..C...A....C....C.....CC.C..G.G........
KY851397 257 SANTANDER CO 2013	.G..A....A........GA....AT....ACAC.......AT.........TG..C...A....C....C.....CC.C..G.G........
JF804029 DB016 CO 2007	.G........................T.....CA....T..A..........TG......A....C.......T..........G..C....A
GQ868552 BIDV3368 SANTANDER CO 1998	............G......A................G...CA.....T....TG.T.............................C.......
DQ364512 CO 1993	.................A.......................A........A.TG......AA.............................T..
